# Two Birds with One Stone: NFAT1-MDM2 Dual Inhibitors for Cancer Therapy †

**DOI:** 10.3390/cells9051176

**Published:** 2020-05-09

**Authors:** Wei Wang, Atif Zafar, Mehrdad Rajaei, Ruiwen Zhang

**Affiliations:** 1 Department of Pharmacological and Pharmaceutical Sciences, College of Pharmacy, University of Houston, Houston, TX 77204, USA; atifzafa@Central.UH.EDU (A.Z.); mrajaei@Central.UH.EDU (M.R.); 2 Drug Discovery Institute, University of Houston, Houston, TX 77204, USA

**Keywords:** NFAT1, MDM2, p53, dual inhibitors, cancer therapy

## Abstract

The tumor suppressor p53 is believed to be the mostly studied molecule in modern biomedical research. Although p53 interacts with hundreds of molecules to exert its biological functions, there are only a few modulators regulating its expression and function, with murine double minute 2 (MDM2) playing a key role in this regard. MDM2 also contributes to malignant transformation and cancer development through p53-dependent and -independent mechanisms. There is an increasing interest in developing MDM2 inhibitors for cancer prevention and therapy. We recently demonstrated that the nuclear factor of activated T cells 1 (NFAT1) activates MDM2 expression. NFAT1 regulates several cellular functions in cancer cells, such as cell proliferation, migration, invasion, angiogenesis, and drug resistance. Both NFAT isoforms and MDM2 are activated and overexpressed in several cancer subtypes. In addition, a positive correlation exists between NFAT1 and MDM2 in tumor tissues. Our recent clinical study has demonstrated that high expression levels of NFAT1 and MDM2 are independent predictors of a poor prognosis in patients with hepatocellular carcinoma. Thus, inhibition of the NFAT1-MDM2 pathway appears to be a novel potential therapeutic strategy for cancer. In this review, we summarize the potential oncogenic roles of MDM2 and NFAT1 in cancer cells and discuss the efforts of discovery and the development of several newly identified MDM2 and NFAT1 inhibitors, focusing on their potent in vitro and in vivo anticancer activities. This review also highlights strategies and future directions, including the need to focus on the development of more specific and effective NFAT1-MDM2 dual inhibitors for cancer therapy.

## 1. Introduction

Cancer remains a leading cause of death and poses a major public health challenge worldwide. Thanks to the impressive progresses made in basic, translational, and clinical biomedical research and development in past decades, we have witnessed a significant improvement in cancer diagnosis and treatment, especially in the fields of targeted therapy and immunotherapy as well as combination therapy with various therapeutic modality. However, there is still an urgent need for the development of more specific effective and safer targeted therapeutics, especially for those devastating solid tumors, such as cancers of the pancreas, liver, and brain, among others. It is generally accepted that cancers stem from an overexpression of oncogenic genes and/or dysfunction of tumor suppressor genes. Among those well-investigated tumor suppressors and oncogenes, the interplay of the tumor suppressor p53 and the murine double minute 2 (MDM2) oncogene has been one of the hottest topics in the research and development of cancer medicine. There are several thousand peer-reviewed publications demonstrating the major oncogenic activities of MDM2 in human cancers, showing that it is not only the best-documented negative regulator of p53 but also exerts p53-independent activities. In the last two decades, there has been an increasing interest in the development of MDM2-based targeted therapies, with hundreds of MDM2 inhibitors being designed and evaluated and several classes of novel MDM2 inhibitors being evaluated in preclinical models and a few entering clinical trials. In this review, we will provide a brief introduction of our current understanding of how MDM2 can be a molecule target, and the current status of representative MDM2 inhibitors under development. We will pay a special attention to the recent findings related to dual targeting of the inflammation pathway and oncogenes and their therapeutic potential for cancer therapy.

MDM2 was originally identified as an oncogene because its overexpression induced tumorigenicity in mouse 3T3 cells [1]. Subsequent studies have shown that MDM2 is overexpressed and amplified in several hematological cancers and solid tumors, and high MDM2 levels are associated with a poor prognosis in patients with cancer [2,3,4]. Further biological and biochemical studies demonstrate that MDM2 is involved in cancer cell growth, apoptosis, cancer cell evasion, metastasis, and resistance to chemotherapy [5,6,7]. MDM2 was initially discovered as a negative regulator of p53 [8,9], but it has subsequently been demonstrated that the MDM2 oncoprotein interacts with many other molecules [5], such as E2 promoter binding factor 1 (E2F1)/retinoblastoma protein (Rb) and X-linked inhibitor of apoptosis protein (XIAP), allowing it to exert a variety of p53-independent effects [10,11]. These characteristics make MDM2 a promising target for the development of anticancer therapies. We, and others, have been interested in developing various classes of MDM2 inhibitors for cancer therapy and other diseases, including antisense oligonucleotides, natural products, and synthetic small molecule compounds [5,10].

**Figure 1:**
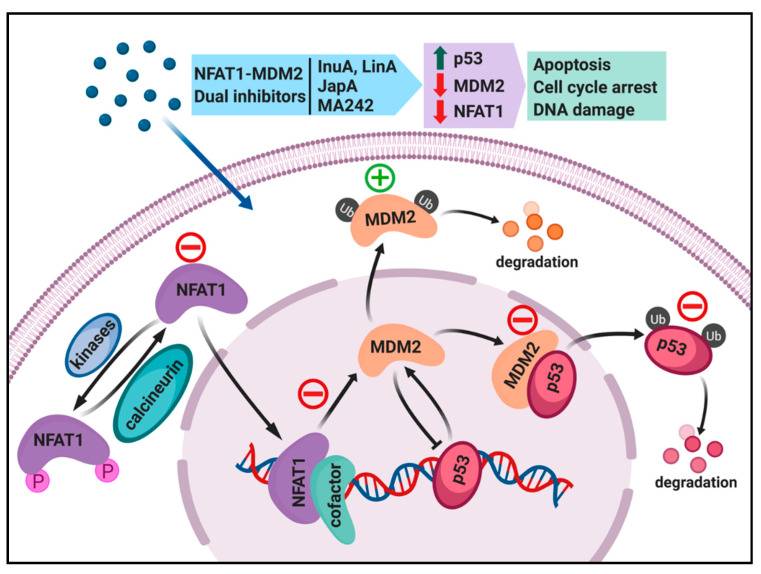
Targeting the NFAT1-MDM2-P53 pathway for cancer therapy. Different classes of NFAT1-MDM2 dual inhibitors can affect different components of this pathway. Lineariifolianoid A (LinA) and Inulanolide A (InuA) inhibit cancer cells by dual inhibition of NFAT1 and MDM2. Japonicone A (JapA) suppresses cancer cells by binding directly to the p53-binding domain of MDM2 and promoting MDM2 auto-ubiquitination, and also through the inhibition of NFAT1-mediated MDM2 transcription and induction of NFAT1 degradation. MA242 induces MDM2 self-ubiquitination and represses NFAT1-mediated transcription of MDM2. The red minus and green symbol indicate inhibition or stimulation induced by NFAT-MDM2 inhibitors.

It has been well demonstrated that there is a clear linkage between inflammation and cancer, prompting us to search for novel cancer therapeutics targeting both inflammation and oncogene. As an example, we recently discovered that nuclear factor of activated T cells 1 (NFAT1) activates the MDM2 oncogene [12]. The NFAT1-MDM2 pathway is activated in various human malignancies, and is associated with a poor prognosis and higher incidence of metastasis [13]. We thus suggest that the NFAT1-MDM2-p53 pathway could be targeted for the treatment of human cancers. In this review, we discuss the oncogenic roles of MDM2 and NFAT1 in cancer cells, and how their interaction affects various steps in carcinogenesis. In addition, this review sheds light on the currently available MDM2 and NFAT1 inhibitors and their effects on cancer cells. We also explain various strategies that can be used to inhibit the NFAT1-MDM2 pathway in cancer cells (Figure 1), and also discuss the dual inhibitors of NFAT and MDM2 with potent in vitro and in vivo anticancer activity.

## 2. MDM2 as a Molecule Target for Cancer Therapy

The major function of p53 is to protect cells from tumorigenesis by inducing cell growth arrest and apoptosis in response to stress signals [14,15,16]. Cellular stress caused by ionizing radiation or chemotherapeutic drugs increases the p53 levels in cells, which leads to a signaling cascade culminating in cell cycle arrest or apoptosis [14,15,16]. The p53 protein upregulates the cyclin-dependent kinase inhibitor 1, p21, in response to DNA damage, which causes cell cycle arrest at G1/S phase and prevents tumorigenesis [17]. Evidence suggests that MDM2 is the most important negative regulator of p53 [8,9,18,19]. Biochemically, MDM2 functions as a ubiquitin ligase and is responsible for the ubiquitination and proteasome-mediated degradation of p53 [18,19,20]. It has also been demonstrated that p53 binds to the P2 promoter of MDM2 and transcriptionally upregulates MDM2 expression [21]. The increased MDM2 then binds p53 and inactivates it by blocking the p53 transactivation domain, thus targeting the p53 protein for degradation [18,19]. This autoregulatory feedback loop helps regulate apoptosis and cell cycle progression [18,19] (Figure 1).

In cancer cells, low levels of p53 are maintained as a result of MDM2 amplification, making these cells able to escape p53′s regulatory control [22]. In addition, it has been found that when MDM2 is present at high levels, it acts as an oncogene [22]. For instance, a study by Fakharzadeh et al. has shown a 50-fold amplification of the MDM2 gene in a tumorigenic mouse cell line (3T3DM), which induces tumorigenesis when experimentally overexpressed in NIH3T3 and Rat2 cells [23]. Post-translationally modified splice variants of MDM2 have also been reported in some cancer cell lines [22]. The MDM2 splice variants lacking a p53-binding domain lead to instability in the MDM2-p53 feedback loop mechanism, thus inhibiting the function of wild-type p53, resulting in tumor growth [22]. It has been estimated that half of human cancers possess a mutant form of p53 [5]. Mutant p53 protein in tumor cells is unable to transcriptionally upregulate MDM2, and as a result leads to high mutant p53 levels in cancer cells [5,24]. Thus, these tumor cells with mutant p53 protein possess a defective negative p53-MDM2 autoregulatory feedback loop [5,24].

Extensive research has been conducted to develop inhibitors of the p53–MDM2 interaction to stabilize p53 and activate apoptosis in cancer cells. Combinatorial library screening has identified several small molecules as MDM2 inhibitors, including spiroxindoles [25,26], nutlins [27,28], isolindones [5,29], and chalone [5,30] derivatives. One such molecule, Nutlin, binds to MDM2 in the p53-binding pocket and activates p53 to induce cell cycle arrest and apoptosis in cancer cells [5,28,29,30]. Several MDM2–p53 binding inhibitors, such as AMG232, RG7112, HDM201, and NVP-CGM097, are currently being evaluated in clinical trials for several human malignancies [31,32]. Our lab has focused on developing new MDM2 inhibitors to induce cell death in cancer cells, regardless of the p53 status of the cells. This quest has led us to develop new MDM2 inhibitors, such as antisense oligonucleotides and siRNA [10], genistein [33], curcumin [34], makaluvamine analogs [35,36,37], and ginsenosides [38,39].

More recently, we have developed a synthetic small molecule MDM2 inhibitor, termed SP141, which not only directly binds to the p53-binding domain of MDM2 but also enhances MDM2 autoubiquitination and proteasomal degradation [40,41], representing a novel class of MDM2 inhibitor with unique mechanisms of action. SP141 has been found to inhibit cancer cell proliferation and induce cell cycle arrest and apoptosis in vitro, and suppresses xenograft tumor growth in vivo, in breast cancer and pancreatic cancer cell lines and animal models, regardless of the p53 status [40,41]. Further, SP141 also inhibits breast cancer cell migration in vitro and prevents lung metastasis of breast cancer in vivo by modulating the expression of epithelial-mesenchymal transition (EMT) markers [41].

Considering the complexity of interactions between MDM2 and its partners in cancer cells, there are ongoing efforts in our lab to further understand the mechanisms of action for MDM2 inhibitors, such as SP141, focusing on the inflammation and oncogene pathways. More recently, we found that SP141 has significant inhibitory effects on β-catenin, a major player in inflammation and oncogenic pathways [42]. Interestingly, MDM2 and β-catenin have both been shown to be overexpressed and constitutively activated in human pancreatic cancer, and the Wnt/β-catenin and MDM2-p53 signaling pathways interact with each other, promoting tumorigenesis and cancer progression and development. The importance of MDM2 and β-catenin molecular targets has been demonstrated by our double knockout experiments [42]. The single silencing of either β-catenin or MDM2 largely reduced the anticancer activity of SP141, while the double knockout of both genes almost completely blocked its activity [42]. This study suggests that a double targeting strategy may be a promising approach to improve the efficacy and safety of MDM2 inhibitors for cancer treatment of advanced pancreatic cancer.

## 3. NFAT1 as a Target for Cancer Therapy

The NFAT family includes five gene products, NFAT1–NFAT5 [43]. All of the NFAT proteins have a Rel homology domain (RHD) and a C-terminal domain [43]. NFAT1-4 possess an N-terminal NFAT-homology domain (NHD), which comprises a transactivation domain and a calcineurin docking site [44]. NFAT5 lacks this calcineurin docking site and is insensitive to calcineurin and calcium [45]. In resting cells, the calcium-sensitive NFAT proteins (NFAT1-4) exist in the hyperphosphorylated form in the cytoplasm [43]. However, upon stimulation, these NFAT proteins are dephosphorylated by calcineurin and translocate into the nucleus, where they activate the transcription of downstream gene targets, thus providing a link between calcium signaling and gene expression [43]. Dysregulation of NFAT signaling is associated with cancer development and progression [43,46]. Studies have shown that NFAT isoforms are overexpressed in several cancer types, including breast cancer [47,48], pancreatic cancer [49], Burkitt’s lymphoma [50], and aggressive T cell lymphoma [51]. Apart from the increased protein levels of NFAT, aberrations in NFAT genes have also been identified [43]. For example, chromosomal translocation in the NFAT1 gene takes place in Ewing sarcoma, where there is formation of an amplified chimeric gene by frame-fusion with the Ewing sarcoma breakpoint region 1 (EWSR1) gene [52,53,54].

The NFAT proteins are involved in cancer cell proliferation, suppressing apoptosis, inducing invasion and migration, and inducing drug resistance through calcineurin-dependent and -independent pathways [42]. For instance, in breast cancer cells, NFAT1 induces MDM2 transcription and increases p53 inactivation, leading to proliferation and an anti-apoptotic environment in cancer cells [12] (Figure 1). In pancreatic cancer cells, NFAT2 is responsible for the displacement of the SMAD family member 3 (Smad3) repressor from the c-Myc gene promoter, leading to the activation of c-Myc transcription [55,56]. NFAT2 also promotes cancer stemness properties in the tumor cell population [57,58]. Constitutive activation of NFAT1 drives breast cancer cell migration and invasion in vitro, apparently via the induction of glypican-6 (GPC-6), cyclooxygenase-2 (COX-2), autotoxin, and prostaglandins [59,60,61]. NFAT5 activation also promotes breast cancer cell migration [62]. It has also been found that NFAT1 stabilizes ski-related novel protein N (SnoN) and mediates transforming growth factor (TGFβ)-induced epithelial-mesenchymal transition in breast cancer cells by downregulating E-cadherin expression and upregulating N-cadherin expression [63]. Although, in the original paper, the authors have not suggested a relationship between NFAT1, MDM2, and E-cadherin, a possible mechanism of E-cadherin downregulation may be caused by NFAT1-mediated upregulation of MDM2. Yang et al. confirmed that E-cadherin is an MDM2 E3 ubiquitin ligase substrate and MDM2 interacts with E-cadherin, resulting in its ubiquitination and degradation [64]. NFAT proteins also play roles in angiogenesis by regulating the transcription of vascular endothelial growth factor (VEGF) receptor 1 in cancer cells [65]. Furthermore, NFAT proteins employ epigenetic means to affect various signaling molecules involved in cancer progression and development [42]. Overall, the crucial importance of NFAT proteins in promoting cancer development and progression suggests that it is an attractive target for cancer therapy.

Inhibition of NFAT signaling has been investigated as a promising strategy for cancer therapy. Several classical inhibitors of calcineurin-NFAT signaling, including cyclosporine A (CsA) and tacrolimus, have shown significant anticancer activity in vitro and in vivo [66,67,68,69]. Mechanistically, tacrolimus and CsA bind to the immunophilin protein and form a drug-immunophilin complex [70]. Since a drug-immunophilin complex directly binds to calcineurin and inhibits its activity, several proteins remain phosphorylated instead of being dephosphorylated, including NFAT, leading to anticancer effects [71,72]. Quercetin, a flavonoid, inhibits tumor growth in a breast cancer xenograft model [73]. Mechanistically, quercetin suppresses the calcineurin/NFAT pathway, inhibiting the expression of VEGF and VEGF receptor 2 (VEGFR2) [73]. Apart from chemical compounds, a peptide termed VIVIT has been developed to inhibit the calcineurin–NFAT interaction, and inhibited NFAT dephosphorylation and nuclear translocation in a model of chronic lymphocytic leukemia [42,74,75]. Although targeting NFAT signaling is a promising approach for cancer therapy, the long-term use of NFAT inhibitors may result in a reduction of immunosurveillance in the tumor microenvironment, subsequently increasing cancer development [76,77]. Thus, it is important that exhaustive studies are carried out to minimize the adverse effects of NFAT inhibitors before their use to target different human malignancies.

## 4. Dual Inhibition of the NFAT1 and MDM2 Pathways for Cancer Therapy

### 4.1. Regulation of MDM2 Expression by NFAT1 and Clinical Relevance

As aforementioned, our lab has demonstrated that NFAT1 regulates MDM2 transcription and activates MDM2 expression [12,33]. The first clue to suggest NFAT1 might regulate MDM2 expression was discovered during our search for natural product MDM2 inhibitors [33]. Genistein, a well-documented natural chemopreventive agent, directly downregulates the MDM2 oncogene, by reducing the levels of MDM2 mRNA and protein in several tested human cell lines, including cancers of the breast, colon, and prostate, and normal cell lines, such as primary fibroblasts and breast epithelial cells. Most importantly, we have demonstrated that such inhibitory effects at both transcriptional and post-translational levels were independent of tyrosine kinase pathways, which have long been believed as the major mechanism of action for genistein.

Further experiments have demonstrated that the NFAT transcription site in the region of the MDM2 P2 promoter is responsive to genistein treatment [33], as first evidence supporting that NFAT1 may regulate MDM2 mRNA expression. Further biochemical and molecular studies have demonstrated that NFAT1 directly binds the *MDM2* P2 promoter, upregulating *MDM2* transcription. Enforced overexpression of NFAT1 in cell lines results in a significant increase in the MDM2 protein level, reducing p53 activation and preventing p53′s response to DNA damage treatment, which suggests that NFAT1′s oncogenic function may be linked to MDM2 activation. The clinical importance of NFAT1 and MDM2 interaction has been demonstrated by several clinical studies [12,39]. Extensive immunohistochemistry (IHC) studies have demonstrated that the levels of both NFAT1 and MDM2 proteins in human hepatocellular carcinoma (HCC) tumor tissues are significantly higher than that of adjacent normal liver tissues, with a positive correlation between the NFAT1 and MDM2 levels being observed in tumor tissues [12].

The next question we asked is if the expression levels of NFAT1 and MDM2 are correlated with the therapeutic outcomes in the clinic. In a recent study [37] aiming at an investigation of the possible correlation between high tumor expression of MDM2 and NFAT1 and a poor prognosis in 254 HCC patients, tissue microarrays (TMAs) were examined, revealing that as high as 60.6% of the HCC cases had high MDM2 expression, and the overexpression of MDM2 was significantly associated with several factors indicating an aggressive clinicopathological course, such as a high alpha-fetoprotein (AFP) level, large tumor size, intensive vascular invasion, and higher tumor stage [37]. Furthermore, HCC patients with high MDM2 expression levels were shown to have lower overall survival (OS) and recurrence-free survival (RFS) than that with low MDM2 expression. Further, multivariate analysis has indicated that the MDM2 expression level is as an independent prognostic factor for the prognosis of HCC patients [37]. Similarly, 57.5% of the HCC cases were shown to have overexpressed NFAT1 and the HCC patients with high NFAT1 expression showed more metastasis and aggressive tumors. HCC patients with high levels of NFAT1 had a shorter OS and RFS, indicating that NFAT1 is an independent prognostic factor for the OS and RFS in HCC patients [37]. More interestingly, MDM2 and NFAT1 were shown to be simultaneously overexpressed in many HCC patients, which was correlated with poor prognosis in those HCC patients as determined by the OS and RFS rates at 1, 3, 5, and 7 years post-hepatectomy [37].

### 4.2. Search for NFAT1 and MDM2 Inhibitors

Since both MDM2 and NFAT1 have independent oncogenic roles in cancer progression and development, simultaneously targeting both proteins represents a novel strategy for cancer treatment. NFAT exists in different isoforms, and each isoform plays a functionally distinct role in human cancer. For instance, NFAT1 and NFAT2 contribute to cancer development, while NFAT3 functions as a tumor suppressor [78,79,80,81]. The currently available NFAT inhibitors may not be adequate for inhibition of the NFAT1-MDM2 pathway in the clinical setting due to their non-selective effects impacting all of the NFAT isoforms [13]. It is thus important to design specific small molecule inhibitors targeting the NFAT1-MDM2-p53 pathway to provide safer and more effective cancer therapy.

There are three main strategies that can be used to inhibit NFAT1 activity: (1) Blocking NFAT1 binding to the P2 promoter of *MDM2*, (2) inhibiting NFAT1 dephosphorylation and/or promoting NFAT1 phosphorylation, and (3) destabilizing the NFAT1 protein [13]. Similarly, there are also three major strategies that can be used to inhibit MDM2 expression and activity: (1) Stimulating MDM2 auto-ubiquitination and proteasomal degradation, (2) blocking the MDM2–p53 or MDM2–other protein interactions, and (3) inhibiting MDM2 expression [13]. Small molecules can be developed to inhibit NFAT1 and MDM2 using the strategies as shown in Figure 1. Ideally, there would be a molecule that can simultaneously act on both targets, providing greater anticancer efficacy and less chance of resistance due to mutations in one of the targets.

### 4.3. Discovery and Evaluation of Dual NFAT1 and MDM2 Inhibitors

Following our initial discovery of genistein as a NFAT1 and MDM2 dual inhibitor [33], there is an increasing interest in identifying more specific MDM2 and NFAT1 dual inhibitors. With our collaborators, we have recently identified several small molecule inhibitors that can simultaneously inhibit both NFAT1 and MDM2, and showed that these exert anticancer effects in vitro and in vivo against several models of cancer (Table 1). Computational structure-based screening leads to the identification of natural product dual NFAT-MDM2 inhibitors, such as JapA [82,83], InuA [84], and LinA [85]. For instance, JapA inhibits breast cancer growth via a dual-targeting mechanism and involves several different effects: (1) The direct binding of JapA to the p53-binding domain of the MDM2 protein and promotion of MDM2 auto-ubiquitination and degradation, independent of p53 [82]; (2) inhibition of NFAT1-mediated MDM2 transcription by suppressing NFAT1 binding to the P2 promoter of MDM2 [83]; and (3) via the induction of NFAT1 ubiquitination and degradation [83]. As JapA analogs, LinA and InuA also suppress breast cancer growth both in vitro and in vivo, and these effects are dependent on their inhibition of NFAT1 and MDM2 [84,85]. Of note, the three naturally occurring compounds may have limited application until a large amount of compounds becomes available through purification from natural sources or a total synthesis.

More recently, we have identified a synthetic small molecule NFAT1 and MDM2 inhibitor, termed MA242, which has been shown to inhibit tumor growth in in vitro and in vivo models of pancreatic cancer and HCC [36,37]. MA242 directly binds both MDM2 and NFAT1 with high affinity, induces their protein degradation, and inhibits NFAT1-mediated MDM2 transcription. MA242 decreases cell proliferation and induces apoptosis in pancreatic cancer cell lines, regardless of p53 status [36]. In the in vivo studies, MA242 inhibits tumor growth and metastasis without any host toxicity, when used alone or in combination with gemcitabine, a clinically used chemotherapeutic agent for pancreatic cancer [36]. Similar results have been recently seen with in vitro and in vivo models of HCC [37]. MA242 profoundly inhibits the growth and metastasis of HCC cells, regardless of the status of p53 in the cancer cells [37]. In brief, the aforementioned efficacy and mechanistic studies provide strong proof-of-principle data to support the translational potential of the NFAT1 and MDM2 dual targeting strategy in future cancer drug discovery and development.

## 5. Conclusions

Thus far, most MDM2 inhibitors under preclinical and clinical development target MDM2–p53 binding and are expected to have little or no effect on cancers without functional p53, including most advanced cancers, such as pancreatic cancer, breast cancer, and HCC. Of note, small molecules that inhibit MDM2–p53 binding indeed show efficacy against p53 wild-type cancers, but most patients who have p53-mutant tumors may have intrinsic resistance to such p53-dependent MDM2 inhibitors. Therefore, p53-independent MDM2 inhibitors may have an implication in a broad spectrum of cancers. As shown in our preclinical studies, targeting the NFAT1-MDM2 pathways is of prime importance to improve the effectiveness of cancer therapy, especially for tumors without functional p53 expression. This approach is based on clinical evidence that NFAT1 and MDM2 are both overexpressed and constitutively activated in different malignancies, and are associated with a high incidence of metastasis and poor prognosis. Our laboratory research has also demonstrated that NFAT1 activates MDM2 transcription via a p53-independent mechanism, and the NFAT1-MDM2 pathway is activated in several cancer cells and clinical tissues. Major efforts are being made to identify new small molecule inhibitors targeting the NFAT1-MDM2 pathway, and to further validate the potential of such inhibitors used alone or in combination in different cancer models.

Interestingly, our findings suggest that NFAT1-MDM2 dual inhibitors may play an important role in the cell cycle check point. As mentioned, p53 participates in multiple cell cycle checkpoints and the importance of MDM2 itself in cell cycle arrest has been largely studied and reviewed [5,6,7]. In addition to downregulating p53, MDM2 interacts with and regulates other molecules, including pRb, E2F1, p21, Cyclin G1, and cyclin-dependent kinase (CDKs), implicating MDM2 in the regulation of cell cycle progression irrespective of the p53 status of cells [5,6,7]. On the other hand, NFAT1 knockout causes altered expression of stage-specific cyclin in lymphocytes, suggesting that NFAT1 plays a major role in regulating cell cycle progression [86,87,88]. In addition, NFAT1 activates the hTERT gene and promotes cell cycle progression in activated peripheral blood lymphocytes [89]. Our recently discovered NFAT1-MDM2 dual inhibitors suppress tumor growth, resulting in cell cycle arrest in different cancer types [36,37,82,83,84,85]. Considering that both MDM2 and NFAT1 play pivotal roles in cell cycle progression, targeting both proteins should generate highly efficient therapeutic agents. In addition, while progress has been made in identifying numerous molecules associated with cell cycle checkpoint control and in evaluating new-generation cell cycle checkpoint inhibitors in preclinical and clinical settings, leading to the first successful Food and Drug Administration (FDA)-approved cancer therapeutic directly targeting cell cycle [90], it is important to note that only a low percentage of patients can benefit from the existing targeted therapies. The new identified NFAT1-MDM2 dual inhibitors may combine well with the cell cycle checkpoint inhibitors, providing new possibilities for improved efficacy of conventional treatment alone.

Recently, hallmark tumor metabolism has become a promising target for anticancer therapeutics [91]. Although several metabolic pathways have been targeted and several clinical trials have been completed, the overall results of the first generation of compounds have been disappointing [91,92]. p53 is recognized as an important metabolism regulator and is involved in the metabolism of amino acids, fatty acids, and glucose; oxidative phosphorylation; reactive oxygen species (ROS) regulation; tricarboxylic acid (TCA) cycle; and growth factor signaling [93]. The role of MDM2 and NFAT1 in metabolism is not clearly understood but several studies have been demonstrated that MDM2 and NFAT1 play critical roles in metabolism regulation and may contribute to tumor progression [94,95,96,97,98,99]. For instance, a recent study demonstrated that MDM2 directly binds to activating transcription factors 3 and 4 (ATF3/4) and regulates a transcriptional activation program involved in amino acid metabolism and redox homeostasis independent of p53 [94,95]. Interestingly, the same research group also found that in the conditions of serine and glycine deprivation and oxidative stress, MDM2 is modulated by pyruvate kinase M2 (PKM2), a key glycolytic enzyme, and controls serine/glycine metabolism and supports cancer growth by regulation of the glutathione metabolism, oxidized nicotinamide adenine dinucleotide (NAD)/reduced nicotinamide adenine dinucleotide (NADH) ratio, and ROS levels, independent of p53 [94,95]. During oxidative stress and hypoxia, increased mitochondrial MDM2 represses NADH-dehydrogenase 6 (MT-ND6) transcription and decreases respiratory complex I activity, resulting in enhanced cancer cell migration and invasion [96]. MDM2 also directly interacts with dihydrofolate reductase (DHFR), a key enzyme in folate metabolism, catalyzes its monoubiquitination and reduces its activity, resulting in regulation of folate metabolism which is intimately connected with DNA metabolism and nucleic acids and protein methylation [97]. Acetyl coenzyme A (Acetyl-CoA) represents a required acetyl donor for lysine acetylation and interacts with many metabolic pathways and transformations [97]. A recent study has shown that Acetyl-CoA promotes cell adhesion and migration in glioblastoma cells. Mechanistically, NFAT1 has been found to mediate acetyl-CoA-dependent gene regulation and cell migration; when acetyl-CoA is abundant, acetyl-CoA induces NFAT1 dephosphorylation and nuclear translocation through the control of Ca2+ homeostasis [98]. Based on novel discoveries and recent progress made in cancer biology in the last few years, future work will be required to elucidate the molecular mechanisms of MDM2 and NFAT1 in cancer metabolism and provide molecular insights to guide future single agent or combinatorial treatment options. Studies of how NFAT1-MDM2 dual inhibitors affect key regulatory pathways should lead to a better understanding of the efficacy of treatments. The efficacy and mechanistic studies will also lead to the identification of prognostic biomarkers for drug response and contribute toward more efficacious and convenient treatment option to patients.

Exploiting metabolic changes to identify anticancer compounds provides novel opportunities for therapeutic intervention in advanced cancer. For instance, oncogenic Ki-ras2 Kirsten rat sarcoma viral oncogene homolog (KRAS) mutations are present in about 30% of all human cancers [100,101]. KRAS has been considered to be undruggable and there are currently no effective targeted therapies for patients with KRAS mutant cancers [100,101]. Recent studies have demonstrated that mutant KRAS-driven cancer cells bypass the glutamine-dependent late G1 checkpoint and arrest in S phase due to the lack of aspartate, presenting exciting opportunities for therapeutic intervention in KRAS-driven cancers [102,103]. The MDM2–p53 interaction inhibitor AMG 232 shows excellent anticancer activity in wild-type p53 HCT116 cells carrying the KRAS mutant [104,105]. A recent study found that the combination of the MDM2–p53 interaction inhibitor (SAR405838) and mitogen-activated protein kinase (MEK) inhibitor (Pimasertib) increases the PUMA and Bcl-2-like protein 11 (BIM) proteins level and leads to cell growth inhibition and apoptosis induction in KRAS mutant and TP53 wild-type non-small cell lung cancers and colorectal cancer models [106]. In another study, the MDM2–p53 interaction inhibitor PXN822 alone or combined with topoisomerase II inhibitor can induce cell death in KRAS-mutated murine pancreatic ductal adenocarcinoma cells, regardless of the p53 status [107]. Interestingly, NFATc1 activation is induced by inflammation and by itself or in cooperation with signal transducer and activator of transcription 3 (STAT3) in pancreatic epithelial cells accelerates carcinogenesis in Kras^G12D^ mice [108]. Pharmacological and genetic strategies to disrupt the NFATc1-STAT3 complex diminishes its tumor-promoting effects [108]. These findings may suggest that dual targeting of MDM2 and NFAT1 is an effective and safe strategy for the treatment of KRAS-driven cancers. The knowledge provided by the recent studies also helps to build the rational for the future design of combinatorial therapies for KRAS-driven cancers.

There is an increasing interest in developing agents that target two or more well-defined molecules as novel anticancer therapeutics. Multiple targeting can be achieved using several agents in combination, or may be achievable using individual agents with multiple targets [109]. Targeting both NFAT1 and MDM2 using a single small molecule is a novel strategy to develop an effective targeted therapy for advanced cancer. Although research related to identifying new dual NFAT1-MDM2 inhibitors is accelerating, more work is needed to elucidate the binding affinity and specificity of the dual NFAT1-MDM2 inhibitors, the precise mechanisms underlying their activity in cancer cells with both wild-type and mutant p53, and also potential immune and other host toxicities that might arise during longer-term or repeated use. Furthermore, high-throughput screening techniques and natural/synthetic combinatorial libraries, along with biochemical and molecular biology approaches, should be implemented for the development of new and effective dual NFAT1-MDM2 inhibitors. Finally, the true potential of such a novel approach relies on future preclinical (pharmacological, pharmaceutical, and toxicological studies) and clinical investigations on the lead compounds and future candidate compounds.

## Figures and Tables

**Table 1:** Selected NFAT1-MDM2 dual inhibitors and in vitro and in vivo anticancer activities.

Compound	Structure	In Vitro and In Vivo Activities
Japonicone A (JapA) [82,83]	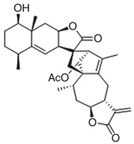	Cell growth inhibition Decreased cell proliferation Inhibition of colony formation G2/M phase arrest Increased apoptosis Inhibition of tumor growth and lung metastasis in breast cancer xenograft models
Inulanolide A (InuA) [84]	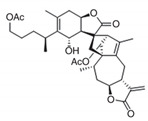	Cell growth inhibition Decreased cell proliferation Inhibition of colony formation G2/M phase arrest Increased apoptosis Inhibition of cell migration and invasion Inhibition of tumor growth and lung metastasis in breast cancer orthotopic models
Lineariifolianoid A (LinA) [85]	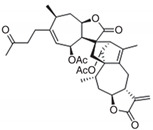	Cell growth inhibition Decreased cell proliferation Inhibition of colony formation G2/M phase arrest Increased apoptosis Inhibition of cell migration and invasion
MA242 [36,37]	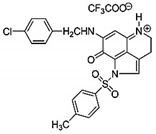	Cell growth inhibition Decreased cell proliferation Inhibition of colony formation G2/M phase arrest Increased apoptosis Inhibition of cell migration and invasion Inhibition of pancreatic tumor growth and metastasis
